# Influence of Microstructure on Fracture Mechanisms of the Heat-Treated AlSi10Mg Alloy Produced by Laser-Based Powder Bed Fusion

**DOI:** 10.3390/ma16052006

**Published:** 2023-02-28

**Authors:** Gianluca Di Egidio, Carla Martini, Johan Börjesson, Ehsan Ghassemali, Lorella Ceschini, Alessandro Morri

**Affiliations:** 1 Department of Industrial Engineering (DIN), Alma Mater Studiorum, University of Bologna, Viale del Risorgimento 4, 40136 Bologna, Italy; 2 Department of Materials and Manufacturing, School of Engineering, Jönköping University, SE-551 11 Jönköping, Sweden

**Keywords:** laser-based powder bed fusion (L-PBF), AlSi10Mg, in-situ tensile test, heat treatment, fracture mechanisms

## Abstract

Few systematic studies on the correlation between alloy microstructure and mechanical failure of the AlSi10Mg alloy produced by laser-based powder bed fusion (L-PBF) are available in the literature. This work investigates the fracture mechanisms of the L-PBF AlSi10Mg alloy in as-built (AB) condition and after three different heat treatments (T5 (4 h at 160 °C), standard T6 (T6B) (1 h at 540 °C followed by 4 h at 160 °C), and rapid T6 (T6R) (10 min at 510 °C followed by 6 h at 160 °C)). In-situ tensile tests were conducted with scanning electron microscopy combined with electron backscattering diffraction. In all samples the crack nucleation was at defects. In AB and T5, the interconnected Si network fostered damage at low strain due to the formation of voids and the fragmentation of the Si phase. T6 heat treatment (T6B and T6R) formed a discrete globular Si morphology with less stress concentration, which delayed the void nucleation and growth in the Al matrix. The analysis empirically confirmed the higher ductility of the T6 microstructure than that of the AB and T5, highlighting the positive effects on the mechanical performance of the more homogeneous distribution of finer Si particles in T6R.

## 1. Introduction

Laser-based powder bed fusion (L-PBF) technology is one of the most commonly used Additive Manufacturing (AM) techniques to produce high-value-added parts in the biomedical, energy, aerospace, and automotive industries. It is receiving significant attention in metal manufacturing as it produces customizable and complex high-performance components with significant weight savings [1,2,3]. AlSi alloys, especially the AlSi10Mg alloy, are perfect candidates to make structural components by the L-PBF process due to their excellent castability, high thermal conductivity, and excellent strength-to-weight ratio achieved by precipitation hardening of the metastable Mg_2_Si phase during and after printing [4,5,6].

The localized thermal radiation of the AlSi10Mg powders leads to rapid heating and cooling rates (10^3^–10^8^ K/s) and non-equilibrium solidification conditions within a semicircular Melt Pool (MP) [7,8], forming a grain-oriented structure and a metastable substructure consisting of supersaturated submicrometer α-Al cells surrounded by a eutectic-Si fibrous network [9,10]. The different strengthening mechanisms deriving from the as-built (AB) microstructure enhance hardness and strength properties but limit ductility and toughness compared with the AlSi10Mg cast alloy [11,12].

The morphology of the eutectic-Si network depends on the thermal history experienced by each region of MP during printing [13,14,15,16]: (i) ultra-fine primary α-Al cells surrounded by a eutectic-Si network in the Melt Pool Core (MPC), (ii) coarser Al cellular structure and thinner eutectic-Si network along the Melt Pool Boundaries (MPBs), and (iii) disrupted intercellular Si network due to the temperatures reached by the underlying layer (3–4 s at 280 °C–560 °C) in the outskirts of the MP (Heat Affected Zone (HAZ)) [4,8]. As Delahaye et al. [13] suggested, the coarser and inhomogeneous Si phase in MPBs and HAZs allows for more straightforward dislocation slip and plastic strain localization, thus limiting the mechanical properties of the AB alloy. Other authors [17,18,19] agreed with these results, underlining the leading role of the inhomogeneous microstructure in the AB alloy’s low ductility and anisotropic mechanical response. As reported in the literature [4,8,17,19], optimized scanning strategies can reduce but not remove the limitations in the mechanical performance of the AB alloy. Therefore, different heat treatments have been investigated in recent years [20,21,22].

Direct artificial aging (AA), also named T5 heat treatment, is adopted on the L-PBF AlSi10Mg alloy to slightly improve the mechanical properties by promoting fine precipitation of the β-Mg_2_Si precursor phases and nano-sized Si precipitates and relieving residual stresses [21,23]. However, the low thermal exposure of the T5 heat treatment (150–240 °C) leaves the MP structure and cellular substructure unaltered, thus leaving problems comparable to the AB alloy [24,25].

Solution and aging heat treatment (T6) can improve the strength-ductility trade-off and promote a more isotropic mechanical behavior [4,20,26]. At the solution (SHT) temperature (T_SHT_) and soaking time (t_SHT_), the interconnected eutectic-Si network evolves into a composite-like microstructure consisting of Si particles embedded into an Al matrix, whose size is purely a function of the T_SHT_ and t_SHT_ [22,27]. However, the coarser microstructure compared with the AB alloy limits the mechanical strength [28,29,30].

Even though several studies on the L-PBF AlSi10Mg alloy microstructure and mechanical behavior have been performed, in the literature, there is a limited systematic analysis of the mechanical damage mechanisms characterizing the different microstructures [4,31]. In particular, the correlation between the eutectic-Si network in the AB and T5 alloys or the spheroidal Si particles in the T6 alloy and the crack propagation in the softer α-Al matrix is still not wholly analyzed [17,19]. In this view, in-situ tensile analysis is the most effective technique for correlating the microstructure with mechanical damage mechanisms during loading [32,33,34,35]. In fact, unlike the analysis on the fracture surface or the metallographic section of samples subjected to mechanical tests, the in-situ technique identifies, in an incontrovertible way, the influence of the microstructural features on the crack nucleation and propagation.

To the best of the authors’ knowledge, only Zhao et al. [32] used in-situ tensile tests to study the fracture mechanisms of the L-PBF AlSi10Mg alloy. In particular, they analyzed three different conditions ((i) AB, (ii) annealed 2 h at 250 °C, and (iii) annealed 2 h at 300 °C), however, they omitted the T6 heat-treated alloy, which is currently the most widely used condition to improve toughness and fatigue performance in high-performance components [4,20,26,36,37]. Therefore, studying the influence of the T6 composite-like microstructure on the fracture mechanisms is crucial. Furthermore, Zhao et al. did not analyze etched specimens in [32], thus precluding a real-time analysis of the influence of the microstructure on fracture nucleation and propagation.

In light of the above, this work aims at studying the relevant mechanical failure mechanisms of the L-PBF AlSi10Mg alloy in the AB and heat-treated conditions, in particular: (i) direct aging (T5), (ii) innovative T6 heat treatment (T6R), and (iii) conventional T6 heat treatment (T6B). The correlation between microstructure, local mechanical properties, and failure mechanisms of the L-PBF AlSi10Mg alloy was investigated by nanoindentation and in-situ tensile tests. The first correlated the local effect of microstructure on mechanical properties; the latter, performed on etched samples, highlighted the role played by the cellular substructure and spheroidal Si particles in the failure mechanisms of the L-PBF AlSi10Mg alloy.

## 2. Materials and Methods

### 2.1. Sample Production and Heat Treatment Designation

**Figure 1:**
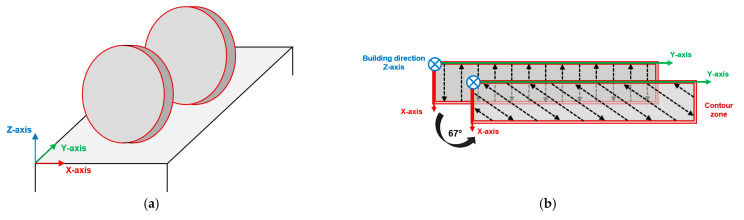
Specimens used in the experimental campaign: printing direction (**a**); scan strategy (**b**).

An industrial SLM500 printing system (SLM Solutions, Lübeck, Germany) produced AlSi10Mg alloy disks (Figure 1a) (diameter Φ = 46 mm and thickness h = 5 mm) adopting the following optimized parameters: 350 W laser power, 1150 mm/s scan speed, 50 μm layer thickness, and 170 μm hatch distance. A heated platform (150 °C) controlled the temperature gradient and reduced the thermal and residual stress during printing. High-purity Ar gas filled the building chamber to reduce the O_2_ content below 0.2 vol.%. A scan strategy consisting of bidirectional stripes of 67° counter-clockwise rotation between subsequent layers and the remelted contour zone was employed to increase the samples’ density and microstructural isotropy (Figure 1b) [38]. As reported in [30], the bulk material’s relative density equals 99.1%. A Glow Discharge Optical Emission Spectroscope (GD-OES, Spectruma Analitik GDA 650, Hof, Germany) verified the samples’ chemical composition, comparing it with the nominal composition of the EN AC-43000 alloy (Table 1). Our previous work [30] reports more information about the powders’ physical and chemical features.

Table 2 reports heat treatment conditions and tensile properties of the L-PBF AlSi10Mg. SHT and AA treatments were conducted in air using an electric furnace with a temperature control of ± 5 °C. Since the sample positioning reduces the temperature inside the furnace chamber, the soaking time was evaluated by achieving the temperature target value for SHT and AA. Two K-type thermocouples were placed next to the specimens to check the temperature’s uniformity during the furnace’s holding time.

### 2.2. Microstructure Characterization and Mechanical Testing

Microstructural and fractographic analyses of the failed samples were performed by optical microscopy (OLYMPUS DSX0010 (Olympus Corporation, Tokyo, Japan) and ZEISS AXIO (Carl Zeiss AG, Oberkochen, Germany)) and Field Emission-gun Scanning Electron Microscopy (FEG-SEM) (LYRA3, TESCAN, Brno, Czech Republic). Cross and longitudinal sections for microstructural analysis were embedded in conductive resin, ground, and finally polished with diamond suspensions up to 1 µm, according to ASTM E3-11(2017) [39]. Then, the samples were etched with Weck’s reagent (3g NH_4_HF_2_, 4 mL HCl, 100 mL H_2_O) [40] according to ASTM E407-07(2015) [41].

Nanoindentation tests analyzed the local effect of microstructure on the mechanical properties. The tests were carried out according to ISO 14577-1:2015 [42] using NanoTest Vantage from Micromaterials equipped with a diamond Berkovich probe (half angle of 65.27°, Young’s Module, Eo = 1141 GPa and Poisson’s ratio, νo = 0.07) and a load speed of 1 mN/s up to a maximum load of 10 mN and a holding time of 5 s at peak load. Sample preparation and data acquisition followed this procedure: (i) sample cutting, (ii) embedding in conductive resin, (iii) metallographic preparation (as previously described), (iv) nanoindentation test, (v) chemical etching of samples using Weck’s reagent, and (vi) image acquisition using OM and FEG-SEM microscopes. Maps of 200 × 200 µm characterized a significant area through equidistant indentations (40 µm). In total, each map elaborated 36 points. Hardness (H) distribution was evaluated using Oliver–Pharr method equations [43]. In particular, H is the ratio between the maximum applied load (P_max_) and the projected contact area at that load (A(h_c_) (Equation (1)):

(1) $$H=\frac{P_{\max}}{A\left(h_{c}\right)}$$

**Figure 2:**
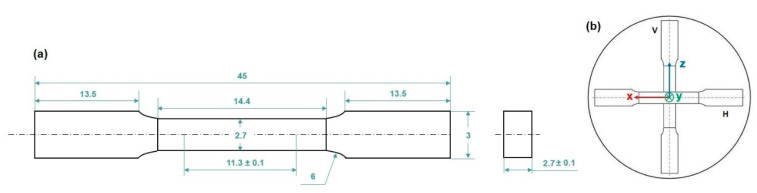
MT sample geometry (dimensions in mm) (**a**). Samples were machined from disks (**b**) in two different directions: parallel (V) and perpendicular (H) to the Z-building direction.

In-situ tensile tests investigated the correlation between microstructure and damage mechanisms. Miniature Tensile (MT) samples were cut using electric discharge machining with a 0.25 mm wire from disks (Figure 1a) in two different directions (Figure 2a) parallel to the Z-building direction (Sample V) and perpendicular to the Z-building direction (Sample H) (Figure 2b).

The tests were performed on H and V samples for each testing condition described previously, as reported in Table 3.

After machining, MT specimens were embedded in resin and polished with abrasive disks, diamond suspensions up to 1 µm, and, finally, with OP-S colloidal silica, according to ASTM E3-11(2017) [39]. This process was performed to preserve the plane parallelism of the samples. EBSD analysis (Hikari Plus, EDAX, Pleasanton, CA, USA) coupled with LYRA3 characterized the crystallographic structure. After extraction from the resin, MT samples were etched with Weck’s reagent according to ASTM E407-07(2015) [41], thus guaranteeing the observation of crack nucleation and propagation within the microstructure during in-situ testing.

**Figure 3:**
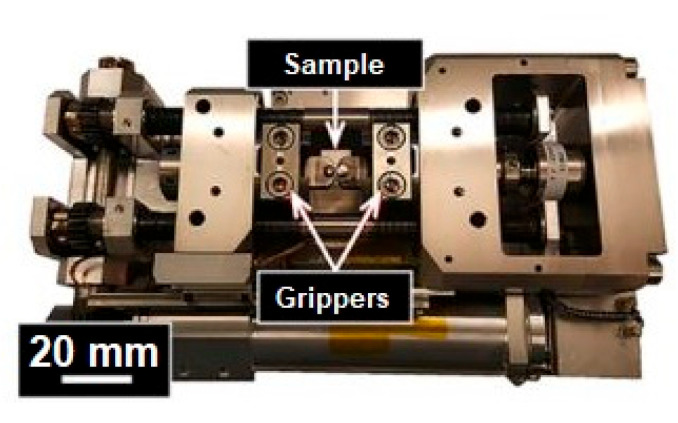
Tensile/compression module for in-situ tensile tests.

In-situ tensile tests were performed according to the ASTM E8-04 [44] on a tensile/compression module (Kammrath & Weiss GmbH, Schwerte, Germany (Figure 3)) placed inside the LYRA3 instrument. The test was characterized by a constant crosshead speed of 0.5 mm/min, which was interrupted at different load steps to evaluate the deformed microstructure and crack evolution.

## 3. Results and Discussions

### 3.1. Microstructural Characterization

#### 3.1.1. OM, SEM, and Nanoindentation Analysis

**Figure 4:**
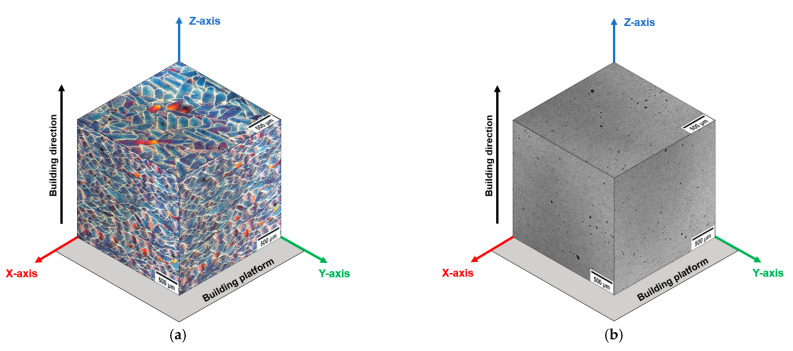
The 3D microstructure of the L-PBF AlSi10Mg alloy in AB condition (**a**) and T6R condition (**b**). Images show the macro-effect of the T_SHT_ and t_SHT_ on the MP structure, as the Authors described in [30].

The scanning strategy adopted in this work promotes a more isotropic microstructure (Figure 4a), enhancing the bulk material’s densification, as described in [7,19,38]. The MPs are distributed randomly across the side and front views (XZ and YZ views), penetrating the previous layers with a variable depth. The microstructure of the top view shows the interception of scan tracks from multiple layers angled at ~67° in a single sample cross-section.

**Figure 5:**
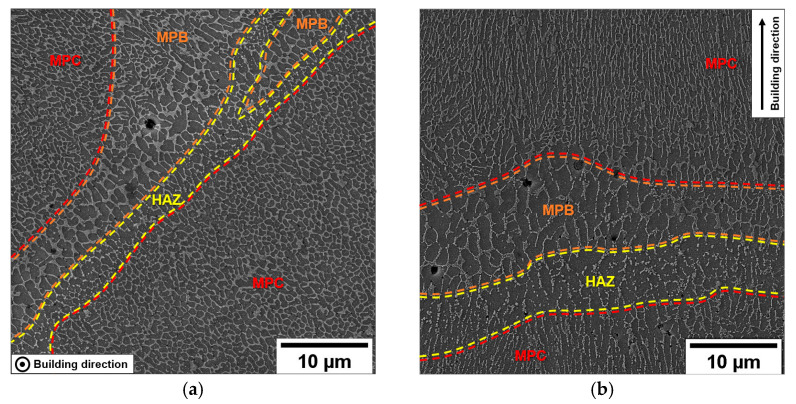
AB cellular substructure in transversal (**a**) and longitudinal section (**b**). Different microstructural zones (MPC, MPB, and HAZ) are distinguishable within the MP.

Due to the high cooling rate, the α-Al phase solidifies into a Si supersatured cellular structure within the MP. At the same time, the residual free Si content concentrates along the α-Al cell boundaries and forms a eutectic-Si network (Figure 5) [45]. The eutectic-Si network morphology changes according to the section view. Observing the section perpendicular to the sample’s building direction, the α-Al cells are distributed in a honey-comb arrangement (Figure 5a). Conversely, in the section parallel to the building direction, the eutectic-Si network appears to be branched and oriented towards the growth direction, with highly elongated α-Al cells parallel to the building direction (Figure 5b). The microstructure of the MPB is comparable in morphology to the MPC, but it is coarser due to different thermal histories, while the Si-rich network within the HAZ appears fragmented, caused by the heat transfer through adjacent layers [13].

**Figure 6:**
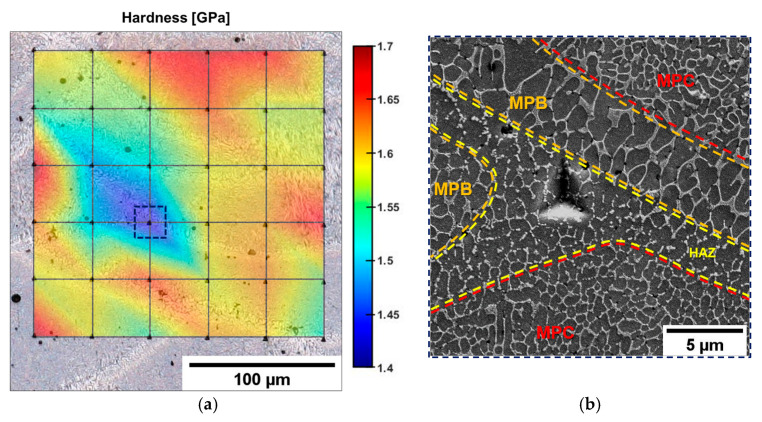
Superimposed OM acquisition of the microstructure and nanoindentation matrix for the AB alloy processed by Matlab^®^ software (version R2022b) (**a**). High-magnification image of the HAZ (**b**), highlighted in (**a**) by the dashed blue box.

The nano-hardness map (Figure 6a) clearly shows the effects of a non-uniform microstructure on the mechanical response. In HAZ (Figure 6b), H values are approximately 1.4–1.5 GPa, lower than the maximum measured value in the MPC (H = 1.7 GPa). This result confirms that the fragmented eutectic-Si network of the HAZ is less effective in inhibiting dislocation slip than the fine (MPC) and coarse (MPB) cellular zones, as described more fully in [46].

**Figure 7:**
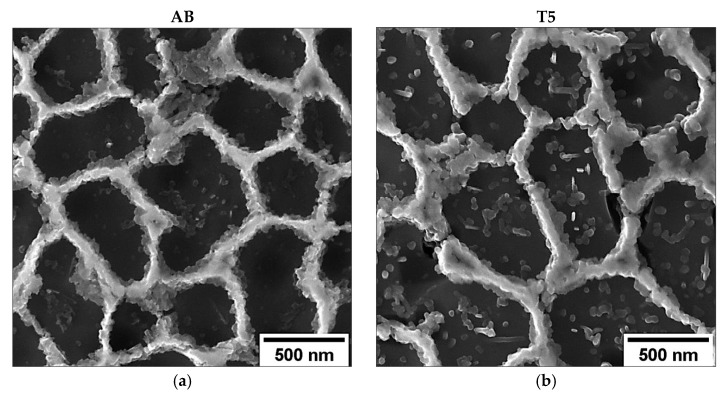

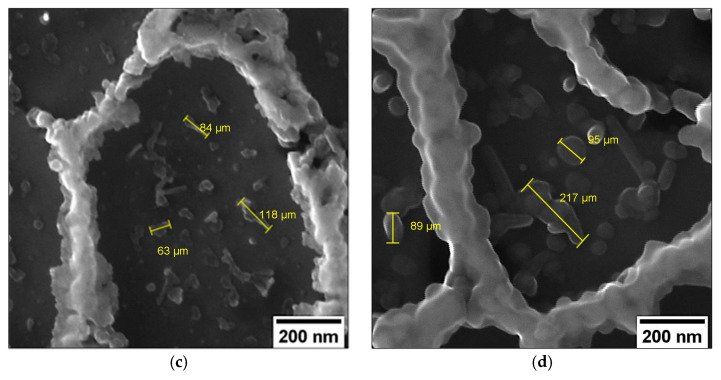
High-magnification FEG-SEM images of the cellular structure in transversal cross-section: AB (**a**,**c**), and T5 (**b**,**d**). Microstructures are comparable, but a higher density of coarser Si nanoparticles is observable in the T5 alloy.

The T5 microstructure does not undergo macroscopic alterations due to the relatively low thermal exposure, keeping the cellular substructure of the AB alloy (Figure 7a,b) unaltered and relieving residual stress [47] (Figure 7). However, the high supersaturation of Si and Mg atoms in the Al lattice leads to the formation and coarsening of β-Mg_2_Si precursor phases and nano-sized Si precipitates inside the α-Al cells [48,49]. Then, nanometric Si precipitates in T5 show larger dimensions than AB (Figure 7c,d).

**Figure 8:**
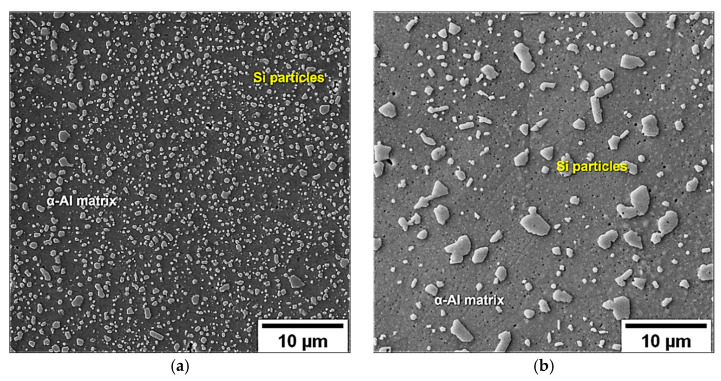
Differences in Si particle size and distribution between T6R (10 min at 510 °C (SHT), and 6 h at 160 °C (AA)) (**a**) and T6B (1 h at 540 °C (SHT) and 4 h at 160 °C (AA)) (**b**).

Instead, the T6R and T6B heat treatments completely erase the MP structure by thermally-activated diffusion processes (Figure 4b). High T_SHT_ leads to the fragmentation and dissolution of the eutectic-Si network and the growth of the initial nano-sized Si particles by the Ostwald ripening mechanism [50]. As a result, the Si network begins to shrink while the nano-sized Si precipitates grow by coalescence, thus forming a homogenous distribution of spheroidal Si particles within the α-Al matrix (Figure 8), whose size increases with increasing the T_SHT_ or extending the t_SHT_ [30]. Consequently, moving from T6R (SHT at 510 °C for 10 min) to T6B (SHT at 540 °C for 1 h), the Si particle average area increases from 0.14 μm^2^ to 0.55 μm^2^, while their density decreases from 1.48 particles/μm^2^ to 0.28 particles/μm^2^ (Figure 8).

**Figure 9:**
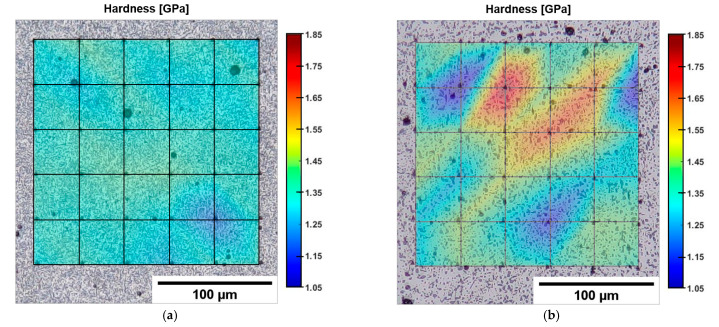
Superimposed OM acquisition of the microstructure and nanoindentation matrix for T6R (**a**) and T6B (**b**) processed by Matlab© software.

The smaller size and more homogeneous distribution of Si particles in T6R lead to a more uniform local mechanical behavior. The indentation map representative of the T6R samples (Figure 9a) shows hardness values between 1.3 GPa and 1.4 GPa with negligible data scatter. In contrast, the T6B shows high differences in hardness between Si particle-free zones (H = 1.1–1.2 GPa) and the zones characterized by large Si particles or Si particle clusters (H = 1.5–1.8 GPa) (Figure 9b).

#### 3.1.2. EBSD Analysis

**Figure 10:**
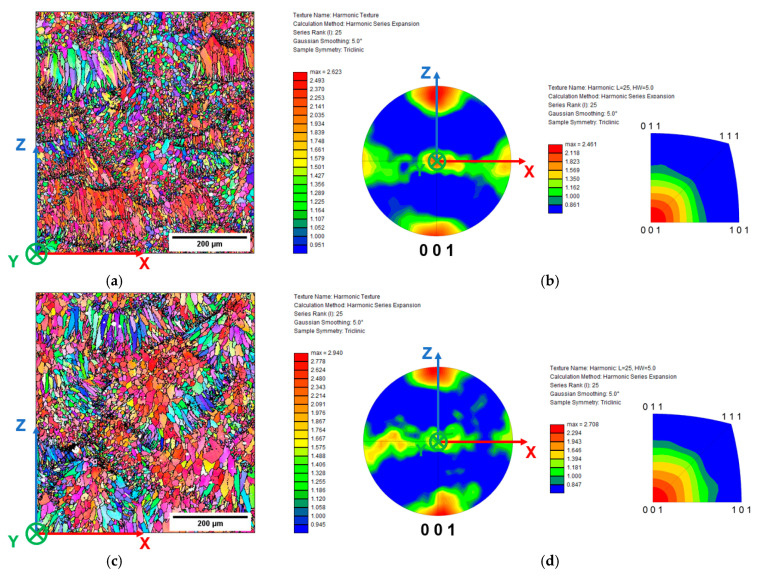

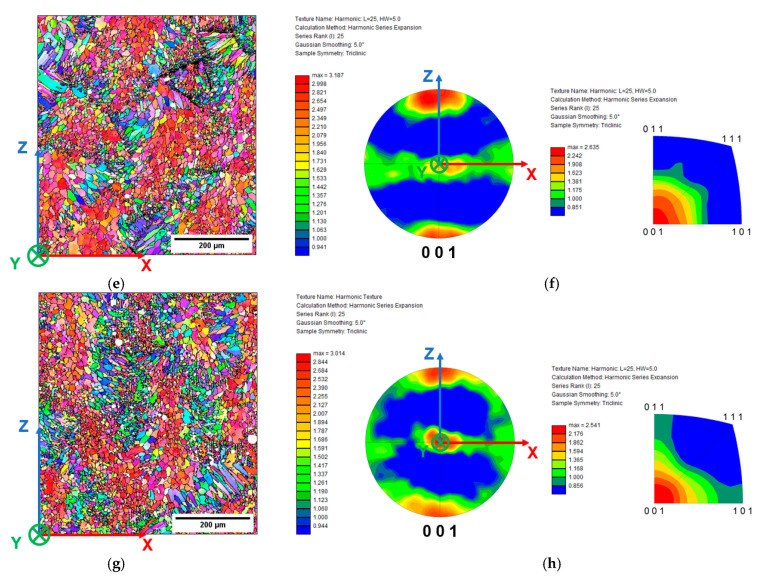
IPF maps, PF, and IPF of the L-PBF AlSi10Mg alloy in AB (**a**,**b**). T5 (**c**,**d**), T6R (**e**,**f**), and T6B (**g**,**h**). In GB maps, the misorientation values higher than 15° are marked by black lines, while lower misorientation was characterized only by a variation in color intensity.

Inverse Pole Figure (IPF), Pole Figures (PF), and IPF maps (Figure 10) provide detailed information on the grain size and texture of the L-PBF AlSi10Mg alloy.

Directional solidification into the MP causes a preferential crystallographic texture oriented along the building Z-direction [51]. As described by Liu et al. [7], the 67° rotation in each layer during printing does not promote significant microstructural differences between the plane parallels to the building direction (XZ and YZ planes) (Figure 4a). Therefore, the EBSD analysis in this work focuses on only one plane, more precisely, the XZ plane.

The IPF map of the AB samples (Figure 10a) shows columnar grains aligned with the centerline of the MP, following the heat flux orientation during the printing process, and smaller equiaxial grains located along the MPBs as a consequence of the partial remelting of the previously solidified layer. In particular, the layer-by-layer deposition strategy influences the epitaxial grain growth and promotes a preferentially oriented crystallographic texture aligned with the heat flow direction [52].

PF and IPF (Figure 10b) reveal the predominant ⟨001⟩ orientation of the columnar grains along the building direction, as evidenced by the red zones at the extremes of the Z-axis of the PF and the high intensities of the orientation ⟨001⟩ in the IPF. The cell solidification mode of cubic materials occurs along the ⟨001⟩ direction, characterized by a higher accommodation coefficient than other crystallographic directions of the FCC structure [7], and promotes the ⟨001⟩ columnar grain texture. Nevertheless, the columnar grains are not perfectly parallel to the building direction, tilting some degrees from the Z-axis due to the influence of the heat flow direction, as described in [52]. In addition, the randomization introduced by the rotating scanning strategy to increase the bulk material’s density induces a texture component along the Y-axis due to the presence of equiaxed grains along the MPBs and a more isotropic microstructure, as also reported by Qin et al. [38] and Paul et al. [8].

The effects of the heat treatment on the grain structure of the L-PBF AlSi10Mg alloy are described in Figure 10c–h. The distribution of the ⟨001⟩-oriented grains in T5 shows comparable characteristics to the AB (Figure 10c,d). In contrast, slight variations characterize T6R and T6B (Figure 10e–h). In particular, T6R and T6B heat treatments did not alter elongated grains’ morphology and texture; the preferential ⟨001⟩ texture orientation along the building direction remains, and the elongated grains’ morphology does not change, even if a higher density of small equiaxed grains enhances the fiber texture component perpendicular to the Z-axis. During SHT, the high content of Si along cell boundaries of the L-PBF AlSi10Mg alloy may induce thermal stresses caused by the thermal expansion coefficient mismatch between Al and Si. The stresses encourage dislocation formation, which can act as nucleation sites for grain recovery [53]. Therefore, the Si particle spheroidization leads to a gradual increase in the number of the sub-grain structures within the columnar grain and a higher number of high-angle boundaries (HAGBs, with a misorientation angle higher than 15°), especially along the MPBs, as described in a previous study [54]. T6B shows the more visible effects: the high temperatures (540 °C) and long soaking times (1 h) promote a significant recovery process [5] observable in a more homogeneous and randomized distribution of the ⟨001⟩ texture orientation along the Z-axis and Y-axis in PF, and weak ⟨011⟩ and ⟨101⟩ texture components in IPF (Figure 10h), as described in [38]. As the T_SHT_ increases, sub-grains can develop from a higher dislocation density due to the differential expansion between Al and Si particles increasing, further emphasized as the Si particle size increases. Therefore, more severe SHT conditions promote larger Si particles, which increase the number of the sub-grain structures measured by the grain-size analysis, as reported by Alghamdi et al. in [5] and the Authors in [54].

### 3.2. In-Situ Tensile Test

#### 3.2.1. Fracture Mechanism Analysis

**Figure 11:**
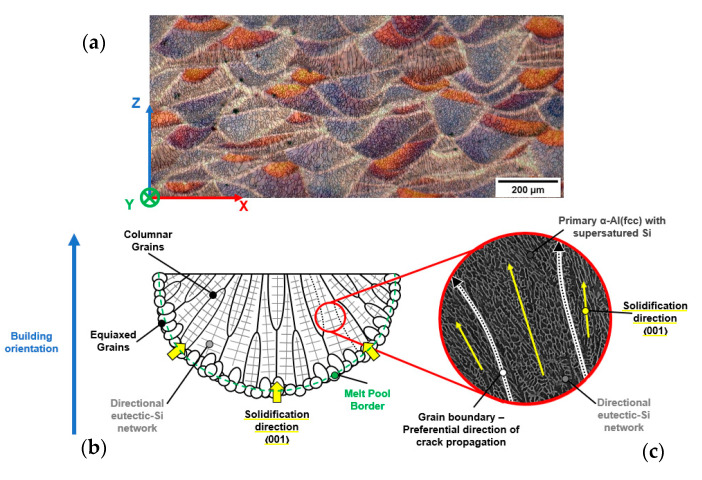
Light optical microscopy (LOM) image of the XZ plane (**a**). Scheme of the preferential orientation of the single microstructural components inside the MP (**b**): misorientation of the cellular substructure between adjacent columnar grains and preferential crack propagation paths (**c**).

In light of the microstructural and EBSD analyses, the hierarchical microstructure of the AB and T5 alloys can be schematized as in Figure 11. The branched cellular structure grows within the columnar grains parallel to the preferred crystallographic directions, aligning with the grain texture orientation (Figure 11b). The overlapping of low resistance areas of the hierarchical microstructure (grain boundaries (GBs), MPBs, and HAZs)) to the eutectic-Si network (Figure 11b,c) promotes crack propagation along predefined preferential directions.

**Figure 12:**
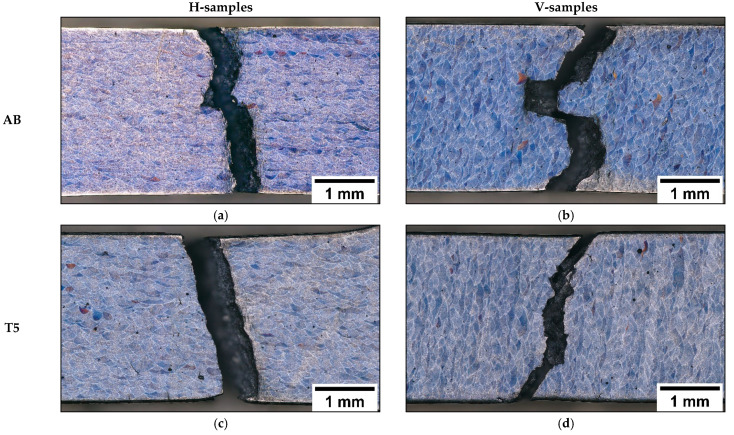
OM images of the fracture area of the in-situ tensile samples: AB (**a**,**b**); T5: T_AA_ = 160 °C t_AA_ = 4 h (**c**,**d**).

AB and T5 show negligible necking (Figure 12a–d). However, they are characterized by different crack propagation paths based on the building direction. In AB-H and T5-H samples (Figure 12a,c), the cracks propagate along the plane perpendicular to the building layers, leading to mixed inter- and intra-MP fracture modes. In contrast, AB-V and T5-V samples (Figure 12b,d) are characterized mainly by fracture propagation along the MPBs. In this case, the inter-layer and inter-MP fracture modes confirm the influence of the load and building orientations on failure mechanisms and mechanical properties, as described in [8,17].

**Figure 13:**
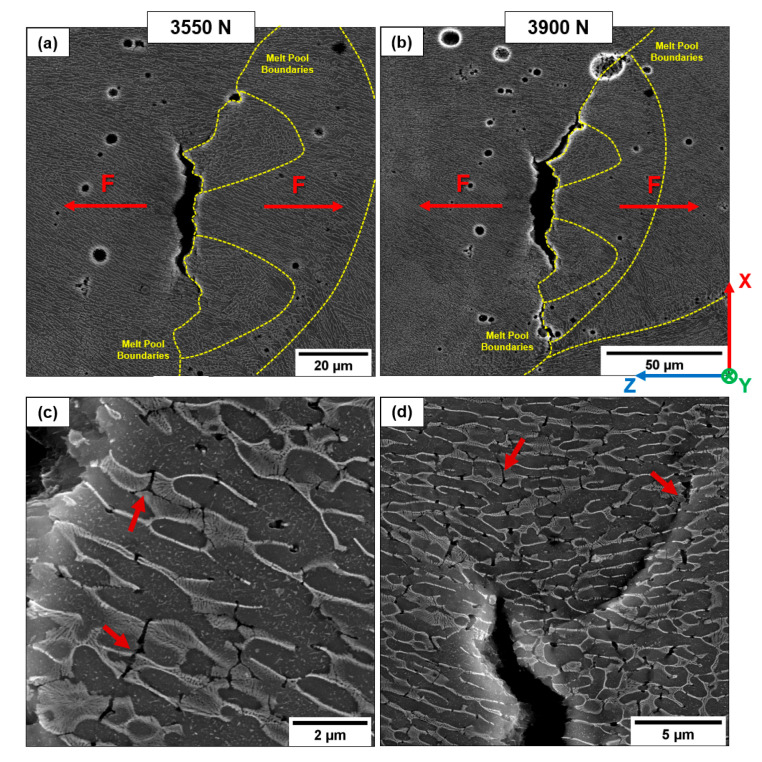
Crack propagation in the T5-V sample. Fracture propagates through the coarser structure (**a**) and along the MPBs (**b**). Details of the severe plastic deformation at the crack tip (**c**,**d**). Red arrows indicate the eutectic-Si network’s breakage and crack formation through the softer Al phase.

In AB-V and T5-V samples, cracks nucleate from a lack-of-fusion (LoF); as the load increases, failure propagation occurs in the coarser and inhomogeneous microstructure of the MPBs and HAZs, characterized by lower strength properties (Figure 13a,b). This mechanism highly reduces the influence of grain texture orientation. The main crack develops by connecting the micro-cracks and voids due to the eutectic-Si network’s breakage and smaller gas pores (Figure 13c,d). The intertwined morphology of the microstructure, generated by the scan strategy and the random distribution of the pores, induces a fracture path not perfectly perpendicular to the load direction (Figure 13b).

**Figure 14:**
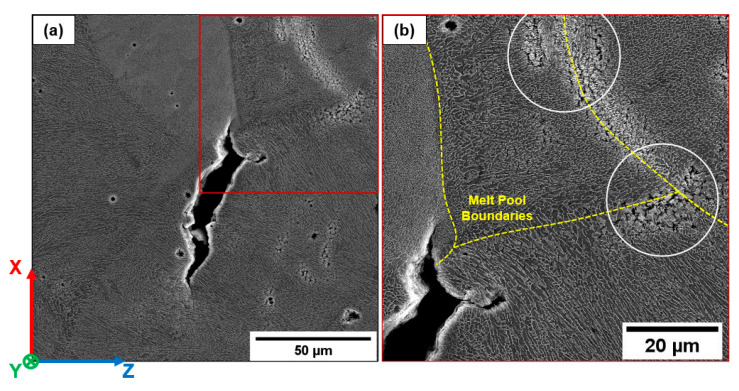
Fracture propagation in the AB-V sample. The crack tip is located in the coarser microstructure of the MPBs (**a**); detail of the fracture propagation (**b**). White circles indicate the void formation at the Si network/α-Al cell interface along the MPBs and HAZs.

Under high local stresses localized at the crack tip, the elastoplastic behavior of the Si and Al phases leads to the rupture of the eutectic-Si network and detachment along the α-Al cell edges (Figure 14). In particular, the void initiation at the Si network/α-Al cell interface is an energetic process where a threshold value of work “W” is necessary to create the crack between the aggregated second phase and the Al matrix (Equation (2)). W depends on three factors, i.e., the surface energy of the matrix γ_Al_, the surface energy of the aggregate second phase γ_Si_, and the interface energy γ_Al-Si_ [13].

(2) $$W\propto {\;\gamma }_{\mathrm{Al}}+\gamma_{\mathrm{Si}}+\gamma_{\left(\mathrm{Al}-\mathrm{Si}\right)}\;$$

The coarser α-Al cell and the thinner eutectic-Si network along the MPBs increase the strain field in the proximity of the second phase due to the difference in the lattice parameters between Si and Al, promoting the loss of coherency between the hard-Si phase and the soft-Al matrix [17]. This condition increases the contribution of the γ_Al-Si_ value, thus reducing the work necessary to nucleate a void at the α-Al/Si interface (Figure 14b). Due to the fragmented Si-eutectic network, the unfavorable stress state condition in the HAZ further emphasizes this phenomenon [4].

**Figure 15:**
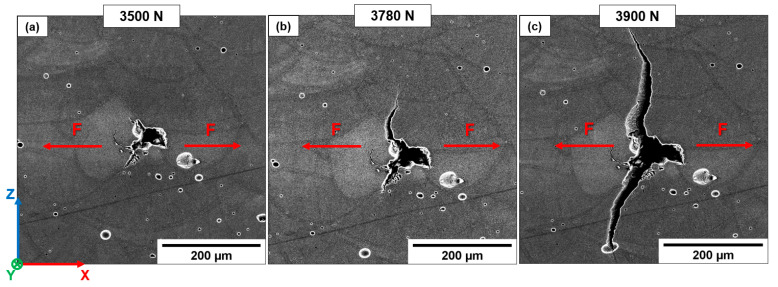
Fracture propagation within the T5-H samples. Large LoF defect is identified at the MP’s intersection zone (**a**). As the load increases, the crack propagates perpendicular to the load direction (**b**), and continues along favorable-oriented planes (**c**).

**Figure 16:**
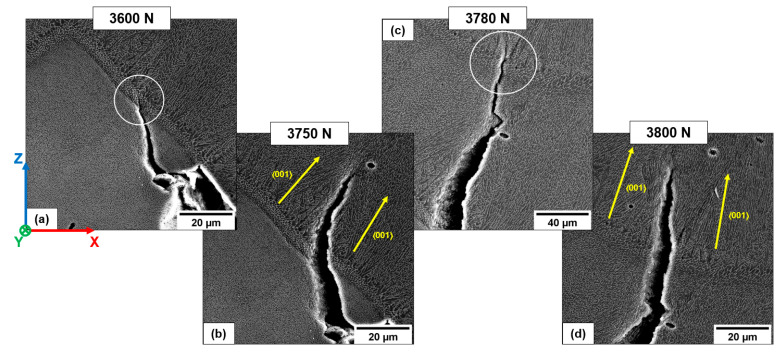
Detail of the fracture propagation (T5-H) reported in Figure 15: crack tip propagates as the load increases (**a**); fracture deviates toward the MPC of the overlying layer (**b**), crosses the MPB, characterized by a coarse microstructure (**c**), and continues toward the MPC of the overlying layer (**d**).

An example of the crack paths occurring in AB-H and T5-H samples is shown in Figure 15a–c. The crack nucleates from an LoF (Figure 15a) and, during the first stages of growth, propagates perpendicular to the load direction along MPBs and HAZs (Figure 15b and Figure 16a). Then, the crack deflects in the MPC and grows along the columnar grain boundaries (Figure 15c and Figure 16).

**Figure 17:**
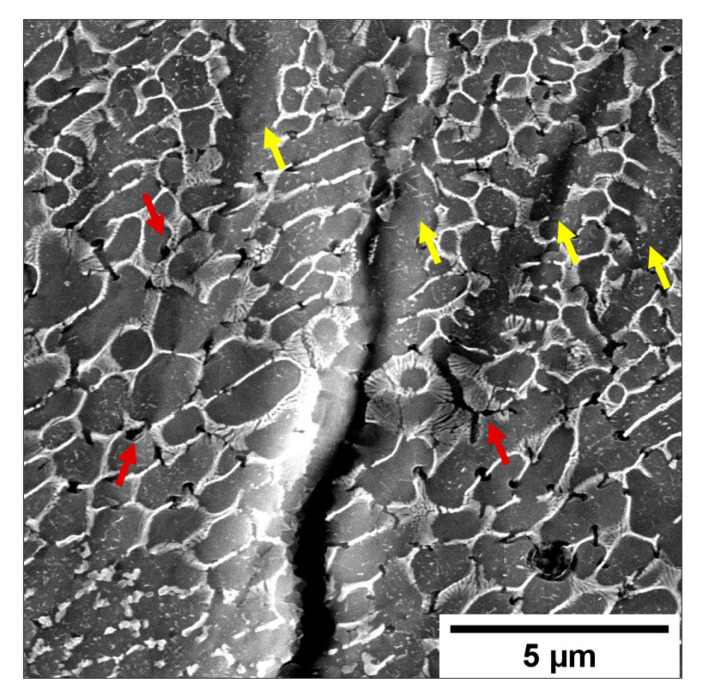
Detail of the intensified stress at the crack tip (white circle in Figure 16c). Red arrows show the formation of voids at the Al/Si interface and the crack through the softer Al phase. Yellow arrows indicate the intense plastic deformation at the crack tip.

A detail of the crack tip (Figure 16c) is reported in Figure 17. It shows: (i) the deformation of the material along the GBs, probably in correspondence with low-angle boundaries [19], (ii) the eutectic-Si network’s breakage and the formation of voids at the interface between the Si and Al phase, and (iii) the growth of the microcracks through the softer Al phase to form small secondary cracks.

**Figure 18:**
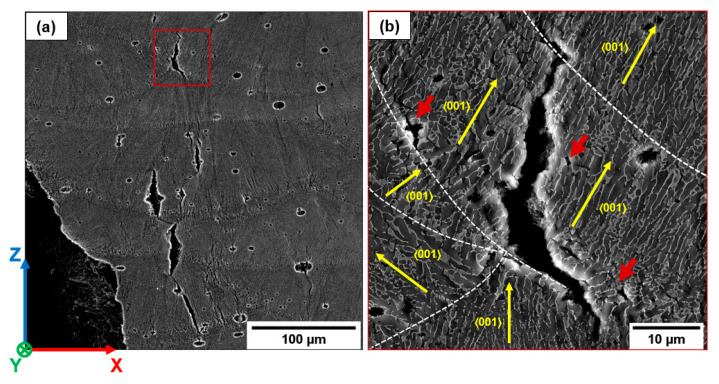
AB-H sample. Crack formation along the GBs close to the fracture surface (**a**). Crack propagation along favorable grain direction within the MPC (**b**). White dotted lines highlight the MPBs. Yellow arrows indicate the solidification direction of the cellular substructure. Red arrows show the formation of voids at the Al/Si interface and cracks through the softer Al phase.

In Figure 18a, cracks close to the fracture surface of the AB-H sample can be observed. Focusing on the secondary crack highlighted in Figure 18b, crack propagation initially occurs perpendicular to the load direction along the HAZ and the MPB, then deviates towards the MPC along the GBs and finally further changes its growth direction following the MPB, with a crack growth mechanism comparable to the T5-H. Furthermore, due to the stress intensification at the crack tip, the fractured eutectic-Si network and voids at the interface between the Si and Al phase are observable.

In view of these observations for AB and T5, the HAZs, MPBs, and GBs represent the preferential crack propagation paths aligned to the maximum shear stress direction. The grain orientation at about 45° to the load direction promotes high local deformation and, consequently, the nucleation of voids and cracks in the softer Al phase in the eutectic-Si network. These findings agree with [14,17]: grain texturing and shear stress direction deeply affect the crack propagation direction.

**Figure 19:**
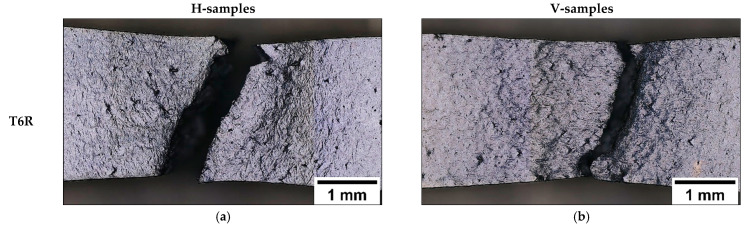

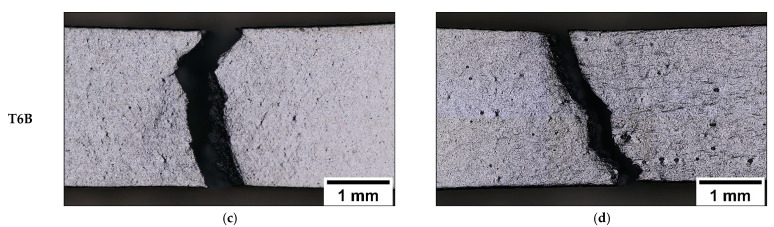
OM images of the fracture area of the in-situ tensile samples. T6R: T_SHT_ = 510 °C, t_SHT_ = 10 min, T_AA_ = 160 °C, t_AA_ = 6 h (**a**,**b**); T6B: T_SHT_ = 540 °C, t_SHT_ = 1 h, T_AA_ = 160 °C, t_AA_ = 4 h (**c**,**d**).

Unlike AB and T5, T6R and T6B (Figure 19a–d) show significant necking in both building directions. While the interconnected eutectic-Si network (AB and T5) fosters damage, the spheroidal morphology of the Si particles delays the void nucleation, which requires higher local plastic deformation at the Al matrix/Si particles interface. This process increases the alloy’s ductility [32]. In particular, T6R (Figure 19a,b) shows higher necking compared to T6B (Figure 19c,d) due to the more homogeneous distribution of finer Si particles [30].

Higher plastic strain in T6B and T6R allows a more accurate observation of grain orientation influence on material deformation. In particular, the T6R-H, T6B-H, T6R-V, and T6B-V samples (Figure 19a–d) exhibit different strain levels based on the influence of building orientation and microstructure on mechanical behavior.

**Figure 20:**
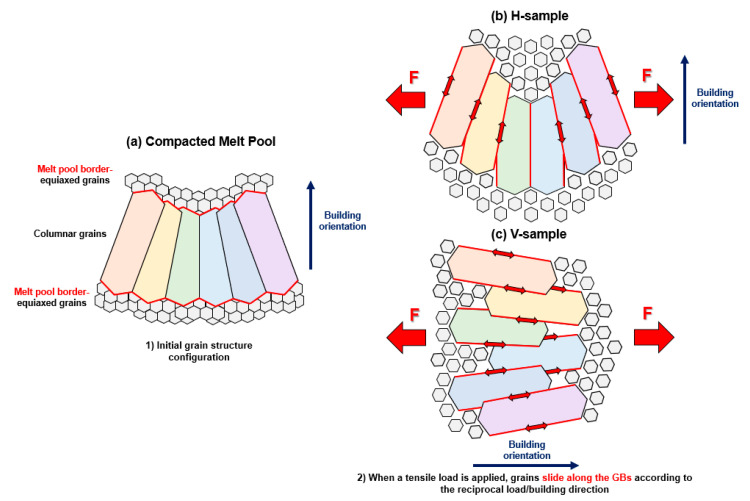
Initial grain structure configuration (**a**). Evolution of the grain structure under tensile load: grain sliding (**b**) and mutual grain spacing (**c**).

**Figure 21:**
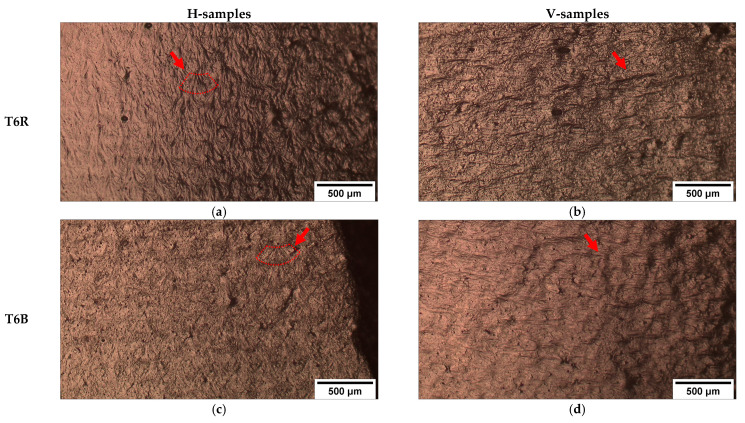
Details of the fracture surface in the T6R (**a**,**b**) and T6B (**c**,**d**) samples. Higher ductility in T6R further emphasizes the effects of plastic deformation. Red arrows indicate columnar grains moving away from each other or sliding along the GBs, while red dotted lines (**a**,**c**) highlight the MP outline.

As schematized in Figure 20, the V-oriented specimens have a more significant strain than the H-oriented ones due to the favorable orientation of the grain within the MP. In particular, a load direction perpendicular to the building direction (Figure 20b) promotes mutual sliding along the GBs in an unfavorable direction to the load. This condition promotes a lower plastic deformation and generates depressions and protrusions in bulk material (Figure 21a,c). For a building direction parallel to the load direction condition (Figure 20c), the applied load encourages the grain to slide along favorably oriented GBs, creating ripples in the bulk material (Figure 21b,d).

**Figure 22:**
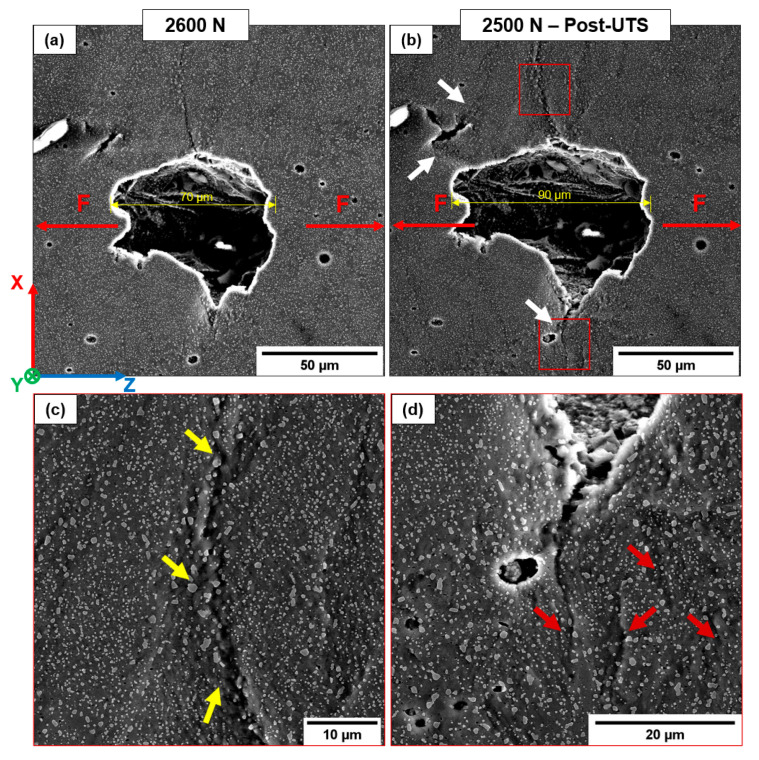
Failure mechanism in the T6R microstructure (T6R-H sample). Defect evolution during in-situ testing (**a**,**b**). White arrows highlight strongly localized deformation in the region between the defects as the tensile load increases. High-magnification images at the crack tip (**c**,**d**). Yellow arrows indicate regions with large Si particle clusters (limited hindering dislocation motion). Red arrows indicate secondary cracks.

T6R has different crack propagation mechanisms compared to AB and T5. In particular, the microstructural homogenization introduced by the T6R heat treatment minimizes differences in the crack propagation path between different building orientations (T6R-H and T6R-V). The composite-like microstructure delays the nucleation of cracks from LoF or gas pores, which undergo significant deformation before crack nucleation and propagation induced by stress intensification. The evolution in size and morphology of a defect during the load application is reported in Figure 22. Even though the applied load exceeds the UTS value (Figure 22b), only small cracks develop perpendicularly to the loading direction from the crack tip. Therefore, the crack propagation only affects low-strength areas characterized by a high density of tiny pores, free-Si particle zone, and large Si particle clusters. In these regions, the highly localized plastic deformation induces the formation of voids at the Si particles/α-Al matrix interface and micro-cracks (Figure 22c,d). However, no crack of Si particles is observed.

The differences in elastoplastic behavior between harder Si particles and the softer Al matrix induce decohesion at the Si particles/α-Al matrix interface and consequent void formation. Furthermore, the high local plastic flow with increasing loading amplifies the inhomogeneous stress state developed around the larger Si particles.

In AB and T5, the leading crack connects the numerous micro-cracks and voids by the eutectic-Si network’s breakage, mainly in the MPBs, HAZs, and GBs. In contrast, the T6R microstructure forces the crack propagation following clusters of larger Si particles or free-Si particle zone. This process leads to a more tortuous and difficult crack path which, together with the increased ductility of the Al matrix, improves the crack propagation resistance of the alloy [25,26,27,28,29].

**Figure 23:**
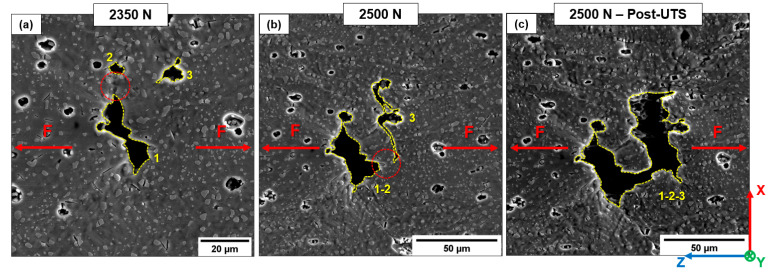
Void coalescence within the T6B-V sample. Separated voids (1, 2, and 3) (**a**); deformation and coalescence of the voids 1 and 2 (**b**); formation of a single large void (**c**). Dashed red circle highlights the zone of intensified stress at the crack tip and junction between the pores.

T6B shows the exact mechanism described for T6R (Figure 23). During loading, pores and LoFs deform and grow while cracks develop at the crack tip, where the highest stresses are present, connecting neighboring voids (Figure 23b). Finally, the coalescence phenomena among adjacent pores lead to forming of a more extensive defect (Figure 23c).

**Figure 24:**
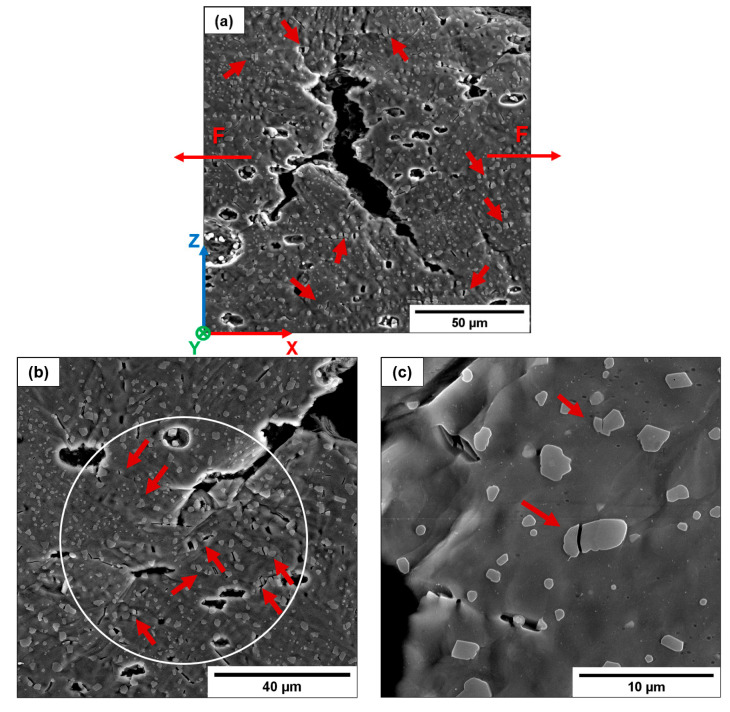
Void coalescence within the T6B-H sample (**a**). Detail of the void coalescence and Si particle cracking at the crack tip caused by intensified stresses (**b**); detail of Si particle cracking (**c**). White circle indicates a highly deformed zone. Red arrows indicate fractured Si particles.

T6B shows a lower plastic deformation and ductility than T6R (Figure 19) due to the presence of coarser Si particles. Under load, they detach easily from the Al matrix, fracturing due to intensified stresses at the crack tip and dislocation pile-up at the Al/Si interface (Figure 24), as reported in [24].

However, in-situ images of T6 samples do not provide appreciable information on the effects of the GB orientation and Si particle distribution on the crack path, as suggested by the fracture surface analysis (Section 3.2.2).

**Figure 25:**
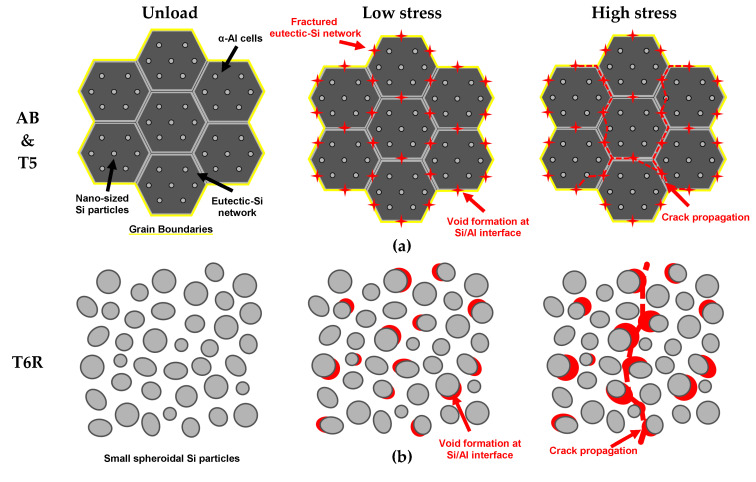

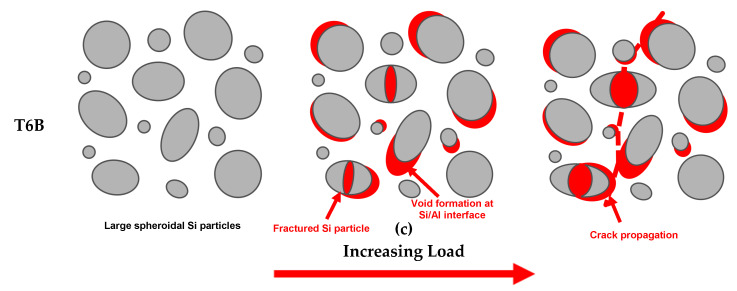
Failure evolution scheme characterizing different microstructures: eutectic-Si network fracturing and void formation and coalescence at Al cell/Si network interface (AB and T5) (**a**); void nucleation at Al matrix/Si particles interface (T6R and T6B) (**b**,**c**) and fractured Si particles (T6B) (**c**), and propagation through shear bands (T6R and T6B) (**b**,**c**).

Figure 25 schematizes the failure mechanisms of the analyzed microstructures. The high strength of AB and T5 is due to the load-bearing capacity of the eutectic-Si network. However, extensive damage nucleates at a low strain on the eutectic-Si network since the Si phase is interconnected and cannot accommodate high strain before failure. Upon loading, the dislocation pile-up in the Al matrix close to the Si-rich interface leads to stacking faults resulting in twinning in the Si-rich cell boundaries and the formation of voids [31]. Once crack propagation starts from defects, the high stresses at the crack tip first lead to the eutectic-Si network fragmentation (Figure 13 and Figure 17), then stress transfers to the enclosed Al cells and undamaged network (Figure 25a). The crack grows by void coalescence mainly occurs in MPBs and HAZs (Figure 14) or along GBs (Figure 16), leading to sample failure.

In contrast, the composite-like microstructure induced by the T6 heat treatment can accommodate high strain before crack nucleation because the Si morphology reduces the stress concentration at the Si/Al matrix interface, leading to plastic flow through the Al matrix and GB sliding (Figure 21). Cracks nucleate from defects and grow by connecting voids at the Si particle/Al matrix interface or failed particles (Figure 25b,c). Therefore, this damage mechanism highly depends on Si particle size and distribution. Small and well-distributed Si particles induce a lower stress concentration than large ones and contain a statistically lower density of internal defects reducing the probability of void formation and Si particle failure. For this reason, fractured Si particles are observed only in T6B (Figure 25c). In contrast, in T6R (Figure 25b), crack growth occurs mainly in correspondence with Si particle clusters or large Si particles.

#### 3.2.2. Fracture Surface Analysis

**Figure 26:**
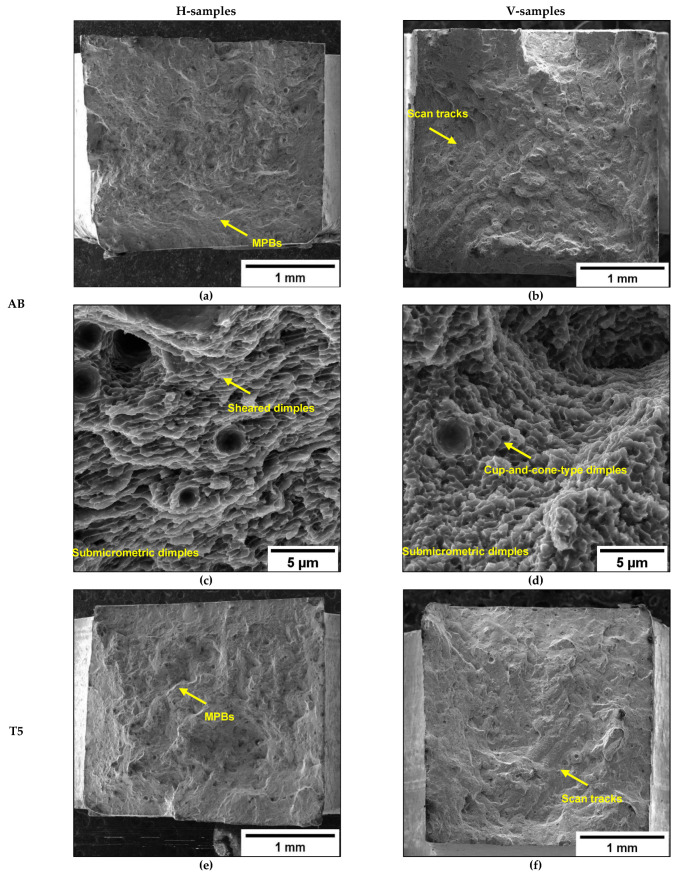

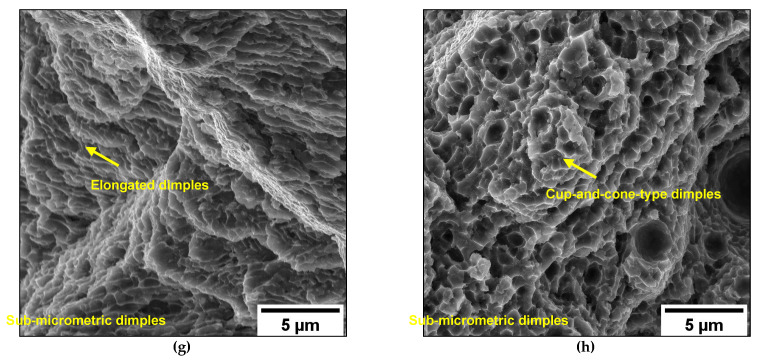
Fracture surfaces at different magnifications of AB-H (**a**,**c**), AB-V (**b**,**d**), T5-H (**e**,**g**), and T5-V (**f**,**h**).

Fracture surfaces of the AB-V and T5-V samples (Figure 26) show step-like features (Figure 26b,f) due to inter-layer crack propagation. During failure, cracks extend mainly along the MPBs and HAZs, showing evidence of the scan strategy on the fracture surface, as observed in Figure 14. Instead, the AB-H and T5-H samples do not show marks of the scan strategy on the fracture surface, thus indicating a more tortuous trans-track fracture propagation mechanism (Figure 26a,e). As reported in Figure 15, the crack propagates both along the MPBs and HAZs and through the MPs.

At higher magnification (Figure 26c,d,g,h), fracture surfaces show shallow dimples induced by plastic deformation and are associated with the detachment of Al cells from the edges of the eutectic-Si network. The Si-rich boundaries impede dislocation motion, thereby increasing the strength of the material. With increasing load, plastic deformation occurs in the Al-matrix, debonding at the Si-rich cell interface. This phenomenon leads to the formation of dimples comparable to the cell sizes. The AB-H and T5-H samples show elongated dimples inclined to the tensile direction (Figure 26c,g), which follow crack deflection in the MPC and propagation along GBs with an orientation of approximately 45° to the load direction (Figure 15c and Figure 16). Cup-and-cone-type dimples, instead, characterize the fracture surfaces of the AB-V and T5-V samples (Figure 26d,h).

**Figure 27:**
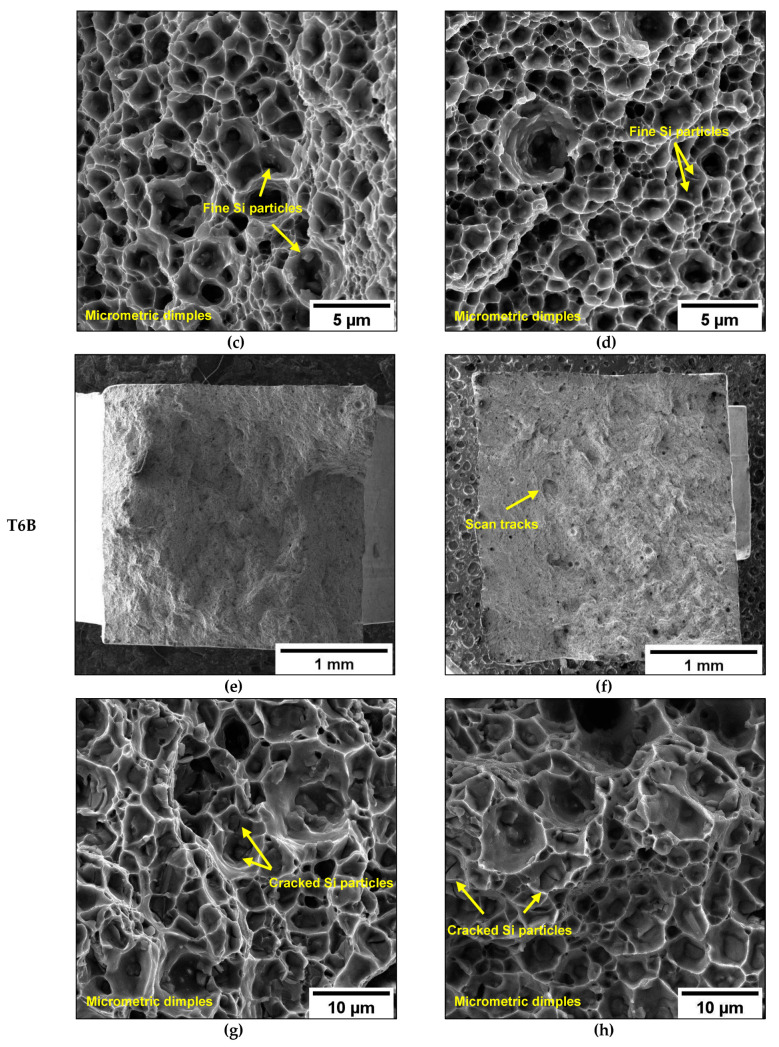
Fracture surfaces at different magnifications of T6R-H (**a**,**c**), T6R-V (**b**,**d**), T6B-H (**e**,**g**), and T6B-V (**f**,**h**).

Even though the T6 heat treatment promotes a microstructural rearrangement and deletes the MPBs and HAZs [36,48], some scan track marks are still observed (Figure 27a,b,e,f), in agreement with Girelli et al. [29]. The different Si content in the AB microstructure between the inhomogeneous and fragmented eutectic-Si network in the MPB and HAZ structures and the high density of small equiaxed grains in MPC (Figure 5) lead to slight differences in the distribution, size, and amount of Si particles [5,55], and play a leading role in crack growth in T6 alloys.

At higher magnification, T6B and T6R show a completely ductile failure mode, characterized by deep dimples (Figure 27c,d,g,h). However, the dimples in T6B (Figure 27g,h) are more extensive and profound than those observed in T6R (Figure 27c,d). Large and failed Si particles and clusters of Si particles induce a concentration of the plastic flow in correspondence with the particles’ borders, resulting in large dimples (Figure 27g,h). In T6R, the homogeneous distribution of small and intact Si particles induces a more uniform plastic deformation, thus leading to finer dimples (Figure 27c,d).

## 4. Conclusions

This study investigated the influence of microstructure on the fracture mechanisms of the L-PBF AlSi10Mg alloy. In-situ tensile tests were performed on AB and heat-treated conditions: T5 (AA at 160 °C for 4 h), T6R (rapid SHT at 510 °C for 10 min followed by AA at 160 °C for 6 h), and T6B (SHT at 540 °C for 1 h followed by AA at 160 °C for 4 h). Failure mechanisms were analyzed to highlight the different microstructures’ role in damage mechanisms, crack initiation, and propagation on samples characterized by different reciprocal load/building orientations. The following conclusions can be drawn:

- AB and T5 alloys show a marked local microstructural anisotropy: lower hardness characterizes HAZs and MPBs, which are less effective in inhibiting dislocation slip than the MPC. The T6 heat treatment homogenizes the microstructure and local mechanical behavior.
- AB and T5 alloys present similar fracture paths, which develop preferentially along the coarser and inhomogeneous zones (MPBs and HAZs). Cracks nucleate from the defects and propagate propagates through the eutectic-Si network, accelerating sample failure. The hard Si-rich phase of the eutectic network limits the plastic behavior, promoting strain localization at the Al/Si interface.
- The T6 alloy can be considered an Al matrix incorporating a hard secondary Si-phase in globular form. The crack propagates from the defects by a void-sheet scenario dominated by the Si particle features, such as shape, size, and distribution, involving the coalescence of the inner pores and voids at the Si particles/α-Al matrix interface.
- The fracture stress of the Si particles and the detachment modes from the α-Al matrix highly depend on Si particle characteristics. Coarser Si particles reduce the T6 alloy’s ductility, as in the T6B alloy, due to the high-stress state at the Si particle/α-Al matrix interface.
- AB and T5 vertical samples exhibit step-like features due to the crack propagation along the MPBs and HAZs. Conversely, AB and T5 horizontal samples do not show significant marks of scan strategy on the fracture surface due to a more tortuous, trans-track fracture propagation mechanism. Shallow dimples formed by the detachment of the Al cells from the eutectic-Si network characterize both conditions.
- Deep dimples characterize the T6 failure mode. In the T6B alloy, coarse and inhomogeneously distributed Si particles form large and very deep dimples, while in the T6R one, the finer Si particles are less prone to fracture, forming smaller dimples. Moreover, the features of some scan track marks are still observed, suggesting that even after the T6 heat treatment, the inhomogeneous Si phase distribution in MPBs and HAZs may play a role in crack growth.

## Figures and Tables

**Table 1:** Chemical compositions (wt.%) of the AlSi10Mg alloy according to EN AC-43000 and nominal chemical compositions (wt.%) evaluated by GD-OES.

Element (wt%)	Al	Si	Mg	Fe	Cu	Mn	Ni	Pb	Sn	Ti	Zn
EN AC-43000	Bal.	9–11	0.20–0.45	<0.55	<0.05	<0.45	<0.05	<0.05	<0.05	<0.15	<0.10
Samples	Bal.	9.74 ± 0.09	0.30 ± 0.03	0.13 ± 0.01	-	0.01 ± 0.00	-	0.01 ± 0.00	0.02 ± 0.01	0.02 ± 0.00	0.04 ± 0.01

**Table 2:** L-PBF AlSi10Mg alloy heat treatment conditions investigated by in-situ tensile test and tensile properties (Ultimate Tensile Strength (UTS), Yield Strength (YS), and elongation to failure (e_f_)). More details can be found in the Authors’ previous work [30].

Condition	Heat Treatment	YS (MPa)	UTS (MPa)	e_f_ (%)
As-built (AB)	-	250 ± 8	447 ± 10	4.1 ± 0.5
T5 direct artificial aging (T5)	AA at 160 °C for 4 h, air cooling	256 ± 3	452 ± 3	4.3 ± 0.6
T6 rapid heat treatment (T6R)	SHT at 510 °C for 10 min, water quenching at room temperature, AA at 160 °C for 6 h, air cooling	251 ± 4	319 ± 6	12.6 ± 0.7
T6 benchmark heat treatment (T6B)	SHT at 540 °C for 1 h, water quenching at room temperature, AA at 160 °C for 4 h, air cooling	221 ± 6	308 ± 7	11.8 ± 0.2

**Table 3:** Heat treatment and building orientation conditions investigated by in-situ tensile test.

Condition	Orientation	
	Horizontal	Vertical
As-built	AB-H	AB-V
T5 direct artificial aging	T5-H	T5-V
T6 benchmark heat treatment	T6B-H	T6B-V
T6 rapid heat treatment	T6R-H	T6R-V
